# Clotrimazole-Loaded Mediterranean Essential Oils NLC: A Synergic Treatment of *Candida* Skin Infections

**DOI:** 10.3390/pharmaceutics11050231

**Published:** 2019-05-13

**Authors:** Claudia Carbone, Maria do Céu Teixeira, Maria do Céu Sousa, Carlos Martins-Gomes, Amelia M. Silva, Eliana Maria Barbosa Souto, Teresa Musumeci

**Affiliations:** 1 Laboratory of Drug Delivery Technology, Department of Drug Sciences, University of Catania, viale A. Doria 6, 95125 Catania, Italy; teresa.musumeci@unict.it; 2 Department of Pharmaceutical Technology, Faculty of Pharmacy, University of Coimbra (FFUC), 3030-548 Coimbra, Portugal; mceuteixeira@ff.uc.pt; 3 CNC—Center for Neurosciences and Cell Biology, University of Coimbra, 3030-548 Coimbra, Portugal; mcsousa@ci.uc.pt; 4 Laboratory of Microbiology, Faculty of Pharmacy, University of Coimbra, 3030-548 Coimbra, Portugal; 5 Department of Biology and Environment, University of Trás-os-Montes e Alto Douro (UTAD), Quinta de Prados, P-5001-801 Vila Real, Portugal; gomes.ma.carlos@gmail.com (C.M.-G.); amsilva@utad.pt (A.M.S.); 6 Centre for Research and Technology of Agro-Environmental and Biological Sciences (CITAB), University of Trás-os-Montes e Alto Douro (UTAD), P-5001-801 Vila Real, Portugal; 7 REQUIMTE/LAQV, Group of Pharmaceutical Technology, Faculty of Pharmacy, University of Coimbra, 3030-548 Coimbra, Portugal

**Keywords:** *Rosmarinus officinalis* L., *Lavandula* x *intermedia* “Sumian”, *Origanum vulgare* subsp. *hirtum*, factorial design, stability, Turbiscan, Lumisizer, DSC, cytotoxicity, MIC

## Abstract

The increasing development of resistance of *Candida* species to traditional drugs represents a great challenge to the medical field for the treatment of skin infections. Essential oils were recently proposed to increase drug effectiveness. Herein, we developed and optimized (2^3^ full factorial design) Mediterranean essential oil (*Rosmarinus officinalis*, *Lavandula* x *intermedia* “Sumian”, *Origanum vulgare* subsp. *hirtum*) lipid nanoparticles for clotrimazole delivery, exploring the potential synergistic effects against *Candida* spp. Small sized nanoparticles (<100 nm) with a very broad size distribution (PDI < 0.15) and long-term stability were successfully prepared. Results of the in vitro biosafety on HaCaT (normal cell line) and A431 (tumoral cell line), allowed us to select *Lavandula* and *Rosmarinus* as anti-proliferative agents with the potential to be used as co-adjuvants in the treatment of non-tumoral proliferative dermal diseases. Results of calorimetric studies on biomembrane models, confirmed the potential antimicrobial activity of the selected oils due to their interaction with membrane permeabilization. Nanoparticles provided a prolonged in vitro release of clotrimazole. In vitro studies against *Candida albicans*, *Candida krusei* and *Candida parapsilosis*, showed an increase of the antifungal activity of clotrimazole-loaded nanoparticles prepared with *Lavandula* or *Rosmarinus*, thus confirming nanostructured lipid carriers (NLC) containing Mediterranean essential oils represent a promising strategy to improve drug effectiveness against topical candidiasis.

## 1. Introduction

Recently, the worldwide incidence of serious systemic infections has extremely increased, most of these due to fungi of *Candida* species, thus causing severe opportunistic infections in immuno-compromised patients, especially those affected by cancer, diabetes and AIDS [1]. Nowadays, the conventional treatment of *Candida* infections requires the use of azoles (clotrimazole, fluconazole, itraconazole, ketoconazole), polyenes (amphotericin B, nystatin) and echinocandins [2,3]. However, different strains of various *Candida* species are developing resistant mechanisms to the treatment with these drugs [1,4]. In addition, conventional antifungal drugs often show toxic side effects to human cells [5,6]. Based on these considerations, researchers’ efforts in developing novel approaches to improve the effective treatment of fungal infection is growing, with a renewed interest in plants and traditional medicine. In this field, increasing attention has been paid to essential oils (EOs) as promising natural compounds for their different activities, such as antibacterial, antifungal, antiviral, antioxidant, anticancer, immune-modulatory, analgesic and anti-inflammatory [7,8]. It has been reported that their broad-spectrum activity against a variety of microorganisms is probably due to the alteration of the membrane and cell wall of microorganisms, with consequent loss of cytoplasmic material [9]. EOs pharmacological activities, mainly related to their complex chemical composition and high concentrations of phenols, make these compounds particularly interesting for both the treatment and the prevention of candidiasis [8,10,11,12]. Nevertheless, EOs low water solubility, high volatility and high instability (oxidation and hydrolysis), strongly limit their practical use in foods, cosmetic and pharmaceutical industries [13,14]. In order to overcome these drawbacks, nanotechnologies have been proposed as a potential valid strategy for EOs encapsulation [15,16,17,18,19]. Recently it has been reported that the encapsulation of *Caryophyllata* EO in SLN improved the rate of microbial killing, reducing the concentration for inhibiting and killing micro-organisms, demonstrating the advantages and efficiency of SLN in combating microbial pathogenesis [16]. SLN containing *Zataria multiflora* EO were successfully prepared, having a mean size of 650 nm, with 93.2% of the essential oil released after 24 h [17]. *Eucalyptus* EO-loaded nanostructured lipid carriers NLC were developed exploiting a synergistic effect between the EO and oleic acid, whose synergism enhanced cell proliferation to confer an advantage in the treatment of wound healing [18]. *Artemisia arborescens* was successfully incorporated into SLN without affecting the in vitro EO antiherpetic activity [19]. Lai et al., also demonstrated the capability of SLN of greatly improving the *Artemisia arborescens* oil accumulation into the skin, and that oil permeation occurred only when the oil was delivered from the control solution [19]. We recently found that *Rosmarinus officinalis*, *Lavandula* x *intermedia* “Sumian” and *Origanum vulgare* subsp. *hirtum* can be successfully used as matrix components and active ingredients of nanostructured lipid carriers (NLC), thus exploiting their relevant anti-inflammatory (or antioxidant) activity, enhancing their biocompatibility and reducing the cytotoxicity of the pure oils [15]. Other authors also reported the possibility to exploit different drug delivery systems for the encapsulation of EOs, such as polymeric poly(varepsilon-caprolactone) (PCL) nanocapsules [20], chitosan nanoparticles [13,21].

Based on these considerations, the aim of the present work was to develop and optimize Mediterranean EOs-loaded NLC for the combined delivery of clotrimazole (CLZ) thus exploiting their potential synergistic effects to the benefit of resistant topical candidiasis treatment. Based on our recent findings, we selected *Rosmarinus officinalis*, *Lavandula* x *intermedia* “Sumian” and *Origanum vulgare* subsp. *hirtum* as intrinsic and active oily liquid component for the optimization of NLC by design of experiment (DOE). The quality by design (QbD) was developed focusing on the quali-quantitative surfactants mixture, for all the selected EOs. NLC were characterized in terms of mean nanoparticles size, polydispersity, structure and long-term physical stability exploiting LUMiSizer^®^ technology. In order to deepen the cytotoxicity effects of EOs on microorganism we also performed calorimetric study on biomembrane models. Furthermore, we aimed to evaluate the biosafety of the selected EOs, pure and loaded into NLC, and to compare the biological effects on two cell lines, namely HaCaT (normal cell line) and A431 (tumoral cell line) selected on the basis of the potential topical application. In vitro antifungal activity against three reference strains of *Candida* spp., namely *C. albicans*, *C. krusei* and *C. parapsilosis* was evaluated.

## 2. Materials and Methods

### 2.1. Materials

Kolliphor RH40 was kindly provided by BASF Italia S.p.a. (Cesano Modena, Italy) while Oleoyl Macrogol-6 Glycerides (Labrafil) was a gift from Gattefossé Italia s.r.l. (Milano, Italy). Hydrogenated Coco-Glycerides (Softisan 100) was purchased from IOI Oleo GmbH (Oleochemicals, IOI group, Hamburg, Germany). Polysorbate 80 (Tween 80), trehalose and chloroform were purchased from Sigma Aldrich Co. (St. Louis, MO, USA). *Rosmarinus officinalis* L., *Lavandula* x *intermedia* “Sumian” and *Origanum vulgare* subsp. *hirtum* essential oils were kindly provided by Exentiae s.r.l. (Catania, Italy). All solvents were for chromatography (LC grade) and were bought from VWR International (Milan, Italy). 1,2-Dimyristoyl-*sn*-glicero-3-phoshocholine (DMPC) was a gift from AVG s.r.l. (Bollate, Milan; Italy). Culture media (Dulbecco’s Modified Eagle Medium, DMEM) and RPMI 1640, fetal bovine serum (FBS), l-glutamine, sodium pyruvate, Versene, trypsin (0.05% trypsin-EDTA), and antibiotics (streptomycin and penicillin) were purchased from Gibco (Life-Technologies, Porto, Portugal). Alamar Blue® reagent was purchased from Invitrogen Life-Technologies (Porto, Portugal). Potato dextrose agar (PDA) medium (Sigma) was purchased from Laborspirit Lda. (Loures, Portugal).

### 2.2. Nanoparticles Production

NLC with *Lavandula* (LNLC), *Origanum* (ONLC) and *Rosmarinus* (RNLC) EOs were produced by high-pressure homogenization (HPH) using the Ultra-Turrax® (IKA, model T25, impeller 10 G, Staufen, Germany), as previously reported [14]. For all formulations, the hot aqueous phase was slowly added to the hot lipid phase. The formulation was mixed for 1 min at 11,000 rpm. An external water bath heated at approximately 70 °C was used to maintain the sample temperature. The hot oil-in-water (*o*/*w*) nanoemulsion was further processed using a high pressure homogenizer (GEA Niro Soavi, model NS1001L2K, PANDA 2 K, Parma, Italy) at 70 °C for three cycles. The final formulation was then cooled to room temperature leading to the lipid phase recrystallization and finally the lipid nanoparticles were formed [15,22]. Drug-loaded NLC with *Lavandula* (CLZ-LNLC) and *Rosmarinus* (CLZ-RNLC), were prepared adding CLZ (0.5% *w*/*v*) to the lipid phase during the preparation procedure.

### 2.3. Design of Experiment (DOE)

In order to optimize NLC composition, an experimental design was performed by using StatSoft7. A full factorial design was employed for the study, based on two factors and three levels (2^3^ full factorial planning). The design was highly suitable for the investigation of quadratic response surface and for generating a second order polynomial model, applied to describe the principal effects and interaction among the identified variables. The independent variables studied in this design such as the concentration of surfactant (A) and the amount of co-surfactant (B) were investigated at three different levels (low, middle and high) and were represented by (−1), (0) and (+1): (i) Surfactant: 3% *w*/*v* (−1), 4.35% *w*/*v* (0), 6% *w*/*v* (+1); (ii) Co-surfactant: 1.5% *w*/*v* (−1), 2.2% *w*/*v* (0), 3% *w*/*v* (+1). ANOVA test was applied to verify the fitted model. Statistical analysis was considered significant when the *p* values were less than 0.05.

### 2.4. Dynamic Light Scattering

Mean particle size (Zave), polydispersity index (PDI) and zeta potential values (ZP) of all unloaded and CLZ-loaded NLC were determined by Dynamic Light Scattering (DLS) using a Zetasizer Nano S90 (Malvern Instruments, Malvern, UK). For measurements, samples were properly diluted (50 µL) in 1 mL of ultra-purified water. Each value was measured at least in triplicate and results are shown as mean ± standard deviation (SD).

### 2.5. EOs Encapsulation Efficiency (EE%)

The amount of EO in the NLC was determined as encapsulation efficiency (EE%) by UV spectroscopy [20], using a UV-Vis 1601 Spectrophotometer (Shimadzu) over wavelengths from 250 to 450 nm (λ max = 260 nm). EE% were calculated after dilutions (1:100) of NLC in methanol, centrifugation and filtration, using the following Equation (1):

EE% = [(amount of EO entrapped)/(total amount of EO used)] × 100(1)

### 2.6. In Vitro Cell Viability Studies

Cell culture, maintenance and manipulation: HaCaT (Human keratinocytes, Cell Lines Services (CLS), Eppelheim, Germany) [23] and A431 (Human epidermoid carcinoma, Cell Lines Services (CLS), Eppelheim, Germany) cell lines were maintained in Dulbecco’s Modified Eagle Medium (DMEM), supplemented with fetal bovine serum (FBS; 10% (*v*/*v*)), penicillin (100 U/mL), streptomycin (100 µg/mL) and L-glutamine (1 mM), in an atmosphere of 5% CO_2_/95% air, with controlled humidity, at 37 °C. Cells were grown, in T25 culture flasks, to near confluence and then were subjected to the action of trypsin, counted (TC10TM, automated cell counter, BIORAD, Amadora, Portugal) and the required volume of cells was re-suspended in FBS-free culture media to give a final density of 5 × 10^4^ cells/mL. Cells were then seeded into 96-well microplates (5 × 10^4^ cells/mL, 100 µL/well), maintained in incubator for 24 h (for adherence), for other details see [15,24].

Cytotoxicity assay: The cytotoxicity of all samples (pure EOs and NLC systems) was evaluated through the Alamar Blue^®^ method. A stock solution of each EO was prepared by diluting each one with DMSO (1:1 *v*/*v*). Before the cell treatment, test solutions were obtained by diluting the stock solution in FBS-free culture media, to give concentrations ranging from 0.001% (*v*/*v*) to 0.5% EO (*v*/*v*). In order to analyze the cell viability of NLC, each formulation was diluted in FBS-free culture media oils. Cells were treated with different concentrations of the pure EOs and NLC for 24 h. After this period, samples were removed and Alamar Blue solution (10% (*v*/*v*) in FBS-free medium) was added (100 µL/well). After 5 h of incubation, the absorbance was read at 570 and 620 nm using a Multiskan EX microplate reader (MTX Labsystems, Bradenton, FL, USA), and the cell viability was calculated using the equations recommended by Alamar Blue manufacturers, as described in [24]. Cell viability is expressed as % of control (non-exposed cells), as mean ± SD, from a set of three independent experiments (each one with quadruplicates). 

### 2.7. Fourier Transform-Infrared (FT-IR) Analysis

Freeze-dried NLC obtained with the addition of trehalose (NLC:cryoprotectant ratio 1:10), freezing suspensions at −80 °C and lyophilizing in a freeze-dryer (Lyph-lock 6 apparatus, Labconco, Kansas City, MO, USA) for 48 h were analysed by Fourier transform-infrared (FT-IR). FT-IR characterizations of pure CLZ and freeze-dried CLZ-loaded NLC were prepared by using different essential oils were performed using a FT-IR spectrophotometer (Perkin Elmer Spectrum RX I, Waltham, MA, USA) equipped with an ATR accessory of diamond Zn/Se. For each sample, 16 scans at a resolution of 2 cm^−1^ were obtained from a wave number 650–4000 cm^−1^, using a speed of 0.50 cm/s and a force gauge of 100 [25].

### 2.8. Accelerated Characterization of Formulation Stability 

An accurate highly accelerated qualitative description of the colloidal suspensions stability was performed by means of the multisample analytical centrifuge LUMiSizer^®^ (LUM GmbH, Berlin, Germany). The instrument employs the patented STEP^®^-Technology, which permits the obtainment of Space- and Time-resolves Extinction Profiles, thus measuring the intensity of the transmitted light during centrifugation, as a function of time and position, over the entire sample length. 

Parallel near infrared light illuminated the entire sample cell and the transmitted light was detected by sensors arranged linearly across the sample from top to bottom. Transmission was converted into extinction by log I/I0 and particle concentration could be calculated. The progression of the transmission profiles contained the complete information on the kinetics of any concentration changes due to creaming, sedimentation, flocculation, coalescence or phase separation [26]. In addition to measuring stability directly and making shelf life prediction, a discrimination between flocculated and nonflocculated dispersions could be performed and particle size distributions could be measured using the highest industry norms and regulations (ISO 13318-2). The analysis was performed as follows: the fresh samples, without prior dilution, were placed in rectangular test-tubes (optical path of 10 mm) and exposed, at 25 °C, to different relative centrifugal forces (RCF): 500 rpm (300 × 30 profiles/s); 2000 rpm (400 × 30 profiles/s); 3000 (350 × 30 profiles/s); 4000 rpm (300 × 30 profiles/s). Such experiment allowed differentiating between various instability mechanisms, at an accelerated rate, in drastically shortened time, and extrapolated results were used to estimate dispersion shelf-life in minutes instead of months. Since instability phenomena, such as creaming or sedimentation, depend on the specificity gravities of the continuous and dispersed phases, which affect preferential particles migration upward or downward, they can be described by Stoke’s law, which allows determining the predictable theoretical value of the instability phenomenon velocity [27]. For this reason, we compared the obtained results with the theoretical model based on Stoke’s law (Equation (2)):

Vs = *g* (*ρp* − *ρ*) *dp*^2^/18 *η*, (2)

where *g* is the gravitational acceleration constant, *ρp* and *ρ* are respectively the densities of the particle and fluid, *dp* is the particle size and *η* is the fluid viscosity. As previously reported [27], the closeness of the measured values of sedimentation/creaming velocities at different RCF to the predictable values based on Stoke’s law, can be exploited to describe the instability phenomena by Stoke’s law. Thus, the obtained data can be used to extrapolate the sedimentation velocity at regular gravity (1 RCF).

### 2.9. Drug Encapsulation Efficiency (EE), Loading Capacity (LC) and In Vitro Release

The amount of the encapsulated CLZ was determined after ultracentrifugation, pellet disruption in Tetrahydrofuran anhydrous (THF), vortex and UV spectrophotometry (Spectrophotometer UV-Vis 1601 Shimadzu, Unilab, Catania, Italy). The encapsulation efficiency (EE%) was calculated by the ratio between the amount of drug entrapped in the nanoparticles and the total amount of drug used for their preparation (Equation (1)). The loading capacity (LC%) was calculated by the ratio between the amount of drug unencapsulated in the nanoparticles and the total amount of lipid used for their preparation (Equation (3)):

LC% = [(amount of encapsulated drug)/(amount of lipid used in the formulation)] × 100(3)

CLZ release from NLC was evaluated by Franz-type diffusion cells (LGA, Berkeley, CA, USA). Before the experiment, the cellulose membranes were moistened by immersion in water for 1 h at room temperature before being mounted in Franz-type diffusion cells. The receptor was filled with water/ethanol (50/50, *v*/*v*) for ensuring pseudo-sink conditions. The receiving solution was constantly stirred and thermostated at 35 °C to maintain the membrane surface at 32 °C. 500 µL of each formulation was applied in the donor compartment on the membrane surface under non occlusion conditions and the experiments were run for 7 days. At fixed time intervals, 200 µL of the receptor phase were withdrawn and replaced with the same volume of receiving solution. Samples were analysed spectrophotometrically to determine CLZ content. 

### 2.10. Biomembrane Model Preparation

Biomembrane models were prepared using the TLE method. Briefly, DMPC (25 mg) was dissolved in chloroform in a Pyrex glass test-tube. The organic solvent was removed at 30 °C on nitrogen stream rotavapor (Rotavapor-M Büchi HB-140, VWR International, Milan, Italy). The formed phospholipid films were dried using a Büchi spray dryer (Büchi TO-51) for 24 h, then hydrated by adding different volumes (999, 995, 990, 980 µL) of isotonic PBS pH 7.4. The tube was alternatively vortexed (Heidolph REAX 2000, VWR International, Milan, Italy) and warmed in a water bath at 40 °C for 3 min twice. The temperature was kept higher than that of DMPC gel-liquid crystal phase transition (24 °C) thus allowing the complete hydration of the phospholipids. In order to evaluate the influence of the two selected EOs (*Lavandula* and *Rosmarinus*) on multilamellar vesicles (MLV), different volumes (20, 10, 5, 1 µL) of each EO and PBS (980, 990, 995, 999 µL) were added to the prepared MLV to obtain 1 mL as final volume. Afterwards, each sample was diluted 1:10 with PBS. The final concentration of the EO in the obtained MLV was in the range of 0.2–0.01% (*v*/*v*). DSC analyses were performed on these final diluted samples. 

### 2.11. Differential Scanning Calorimetry

DSC studies were performed on diluted MLV as above described. The prepared samples were sealed in an aluminium pan and submitted to DSC analysis to determine the influence of the selected EOs on the thermotropic parameters of phospholipid bilayers used as biomembrane models. A Mettler Toledo DSC 1 STARe system equipped with a PolyScience temperature controller (PolyScience, Niles, IL, USA) was used to perform calorimetric analysis. The detection system was an HSS8 high sensitivity sensor (120 gold–gold/palladium-palladium thermocouples) and a ceramic sensor (Mettler Full Range; FRS5) with 56 thermocouples. The signal time constant was 18 s and the digital resolution of the measurement signal was less than 0.04 μW. Calorimetric resolution and sensitivity determined by TAWN test were respectively 0.12 and 11.9. The sampling rate was 50 values/s. The sensitivity was automatically chosen as the maximum possible by the calorimetric system, and the reference was an empty pan. The calorimetric system was calibrated, in temperature and enthalpy changes, by using indium by following the procedure of the DSC 1 Mettler TA STARe instrument. The reference was an aluminium pan containing 100 μL isotonic PBS (pH 7.4). Each sample was submitted to heating and cooling cycles (two times), in the temperature range 5–55 °C, at a scanning rate of 2 °C/min for heating and 5 °C/min for cooling. We evaluated the results of the second heating. Thermotropic parameters were calculated with Mettler STARe Evaluation software system (version 15.01) installed on an Optiplex 3020 DELL. Pure EOs and NLC were studied with DSC in the same range of temperature used for MLV investigations, results are reported in Supplementary Figure S1.

### 2.12. In Vitro Antifungal Susceptibility Test

The in vitro antifungal susceptibility test for the selected CLZ-NLC formulations was performed based on the Clinical and Laboratory Standards Institute (CLSI) reference protocols M27-A3 broth microdilution (BMD) method [28] for yeasts, with minor adaptations, using sterile, disposable, multiwell microdilution plates (96 U-shaped wells). The test was performed using three reference strains of *Candida* spp., namely *C. albicans* ATCC 10231, *C. krusei* ATCC 6258 and *C. parapsilosis* ATCC 90098. For comparative purposes, in addition to the CLZ-LNLC and CLZ-RNLC formulations, LNLC and RNLC systems were also tested, as well as free EOs (L and R) and free CLZ. Briefly, inoculum suspensions were prepared at appropriate densities in RPMI 1640 broth (with L-glutamine, without bicarbonate, and with the pH indicator phenol red) from 24h PDA cultures. Stock solutions of free EOs, CLZ and NLC formulations were prepared in MilliQ water using Tween 80 as a co-solvent, sterilized by filtration and then diluted in RPMI 1640 broth. Free EOs and all NLC formulations were tested at different concentrations in the range 0.03125–2% *v*/*v* while free CLZ was tested in the range 2–128 µg mL^−1^, determined based on the combined information available in KnowledgeBase (http://antibiotics.toku-e.com) for the three strains. NLC-free growth controls, sterility and Tween 80 wells, were also included. The microplates were incubated for 48 h at 35 °C.

After incubation, the yeast growth was screened by OD measurement at 525 nm and the content of first five dilutions tested of each formulation was transferred into to PDA plates and incubated for a new 24 h period at 35 °C. Minimum inhibitory concentration (MIC) values, considered as the lowest concentration of each solution causing full growth inhibition, and minimum lethal concentration (MLC) values, considered as the lowest concentration of each solution causing fungal death, were determined after visual counting of yeast colonies.

### 2.13. Statistical Analysis

All plotted data were presented as a mean of three different experiments ± SD. Differences between the calculated means of each individual group were determined by one-way ANOVA test using the statistical tools available in GraphPad Prism 6 software. A value of *p* < 0.05 was considered statistically significant. Significant differences (*p* < 0.05) between EO and respective NLCs in cell viability results for the two cell lines were obtained by using two-way ANOVA with a Tukey post-hoc test. 

## 3. Results and Discussion

### 3.1. NLC optimization for EOs Encapsulations (DoE)

**Figure 1:**
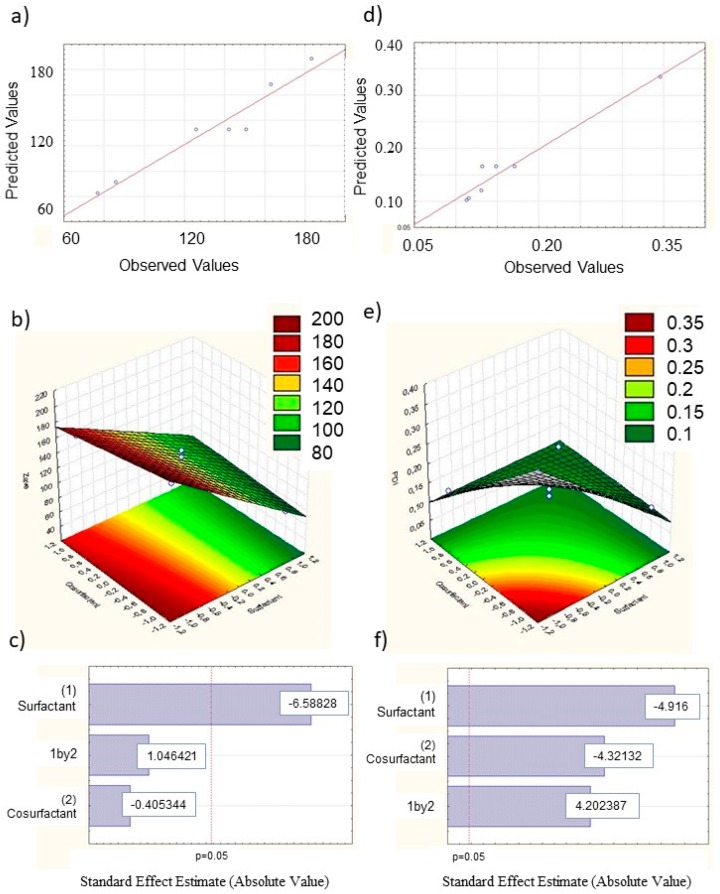
Design of Experiment (DoE) results: observed *vs* predicted values for Zave (**a**) and PDI (**d**), respectively; Response surface plots for Zave (**b**) and PDI (**e**); Pareto Chart of standardized effects for Zave (**c**) and PDI (**f**).

In this work, we exploited the Design of Experiments (DoE) as a tool of the Quality by Design approach (QbD), with the aim to develop optimized formulations in terms of particles with small size and high homogeneity. In particular, a full factorial design was developed with two independent variables (x1, x2) and two levels (−1, +1) (Table 1 and Table 2, Figure 1).

As previously reported in literature, the QbD led to a huge amount of information, saving both time and costs of the experiment [29,30].

The results of DOE experiments on NLC prepared with *Lavandula* x *intermedia* “Sumian” showed that nanoparticle features are affected by the amount of surfactant and cosurfactant. In particular, a wide variation in response was obtained, with Zave values ranging from 70 nm to 200 nm, while PDI ranged from a minimum of 0.11 to a maximum of 0.35. Coefficient regression values were found to be higher for Zave when compared to PDI. The highest coefficient value found for the surfactant (β1) demonstrates a very high significant effect (Table 1). 

Furthermore, the negative values of the calculated regression coefficients for both independent variables confirms the occurrence of a negative relation between both compounds and the dependent variable Zave. This result is in accordance with previous findings, confirming that a perfectly balanced amount of surfactants would decrease the surface tension at the interface between the aqueous and lipid phase, thus determining the formation of well-stabilized smaller particles [31,32].

It is worthy to note that, surprisingly, by decreasing the amount of co-surfactant, an insignificant effect was observed on mean particle diameter. Thus, the exerted energy during nanoparticle production would be enough for coating nanoparticles’ surface, even using less co-surfactant, thus resulting, contrary to previous literature findings, in reduced sized nanoparticles [31,32,33,34,35]. Interestingly, our results show that both the independent variables and their interaction significantly affect (*p* < 0.05) nanoparticles homogeneity, as shown in Figure 1.

Similar findings were observed in the response surface plot obtained for the second order polynomial equation of NLC prepared with *Rosmarinus* and *Origanum* (data not reported).

The above considerations highlight that the amount of the surfactant mixture was able to significantly affect the physicochemical properties of EOs NLC but not the EE% of the EO, that was very high (~96%) for all EOs, in accordance with previous findings [18,20]. Furthermore, our findings allowed us to select the ratio 4:1 of the mixture Kolliphor/Labrafil, as the ideal surfactants combination leading to the formation of NLC with small particles (<100 nm) and a very broad size distribution (PDI < 0.15), related to a very stable and promising system, as reported by Patravale et al. [36].

### 3.2. Biocompatibility of EOs and NLC-EO Using Human Cell Lines

The beneficial effects of EOs on dermatological applications have been established for a long time, although most available information comes from ethnopharmacological surveys. As example, the use of *Lavandula* EO, has been reported to have positive effects in the treatment of eczema, dermatitis, in the improvement of skin wound healing with less scars and has been recommended for the treatment of skin diseases such as psoriasis [37]. 

**Figure 2:**
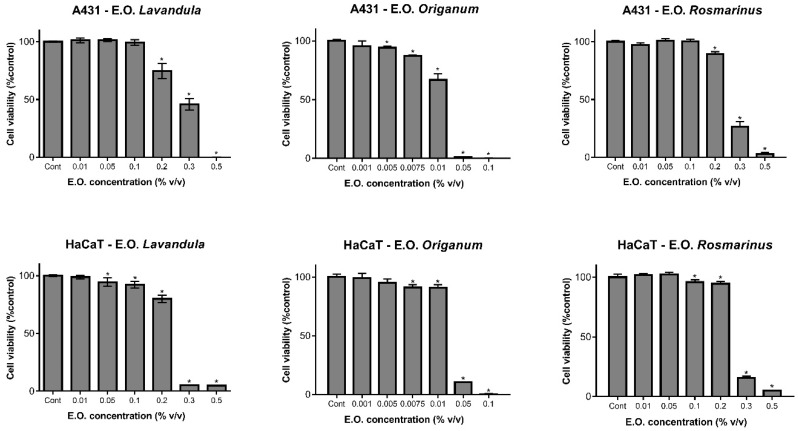
Cell viability results for A-431 (at the top) and for HaCaT (at the bottom) cells exposed with various concentrations of essential oils from *Lavandula*, *Origanum* and *Rosmarinus* (as denoted). Results are presented as mean ± SD (*n* = 3 independent experiments). * Significance for *p* < 0.05.

In this study we aimed to evaluate the biosafety of the three EOs of the Lamiaceae Family (*Lavandula*, *Origanum* and *Rosmarinus*) as well as of the respective prepared NLC (LNLC, ONLC, RNLC), and to compare their biological effects on two cell lines, namely HaCaT (a normal cell line) and A431 (a tumoral cell line). For that, HaCaT and A431 cells were exposed to a set of concentrations of EOs (see methods for details) during 24 h, and cell viability was accessed using the metabolic indicator Alamar Blue. HaCaT and A431 cell viability results (expressed as percentage of control (non-exposed cells)) of the treatment with the pure EOs are reported in Figure 2. 

Results showed that pure *Lavandula* and *Rosmarinus* were safer on both cell lines compared to *Origanum*. In particular, IC_50_ values obtained for *Lavandula* were 0.228 ± 0.004 (% *v*/*v*) and 0.274 ± 0.006 (% *v*/*v*) which are ~10-fold (HaCaT) and ~23-fold higher than those obtained for *Origanum* (Table 3).

As reported in previous literature, different *Origanum* species, including *Origanum vulgare* subsp. *hirtum*, showed high levels of cytotoxicity against cell lines. In particular, the treatment on four different cell lines (Vero, African green monkey, kidney; Hep-2, human epidermoid larynx carcinoma; RSC, rabbit skin; and HeLa, human cervix epitheloid carcinoma) showed IC_50_ values of about 0.0027% (*v*/*v*) for all cells [38], which are lower than those obtained in this work (Table 3). However, *Origanum* pure EO cytotoxic effect was higher (almost two-fold) in the treatment against the tumoral cell line compared to HaCaT, a normal keratinocytes cell line (Figure 2, Table 3). Results of the treatment with pure *Lavandula* and *Rosmarinus* were similar in both cell lines (Figure 2), with any reduction of cell viability observed at the concentration of 0.05% *v*/*v*. IC_50_ values obtained for *Rosmarinus* EO were similar to those obtained for *Lavandula* (Table 3). In addition, these results are in agreement with our previous findings on the cytotoxicity of similar NLC tested on RAW 264.7 cells [15].

**Figure 3:**
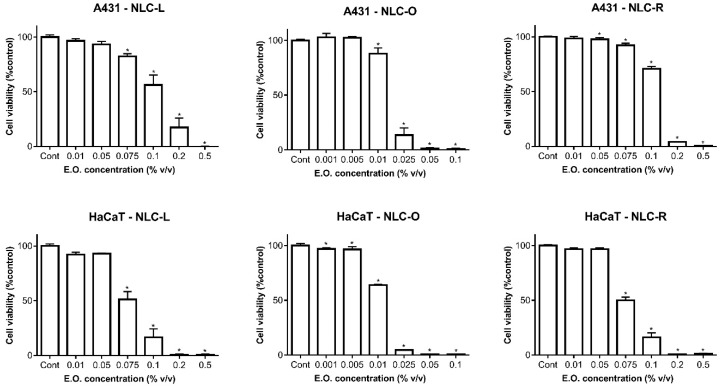
Cell viability results for A-431 (at the top) and for HaCaT (at the bottom) cells treated with NLC-L, NLC-O and NLC-R. Results are presented as mean ± SD (*n* = 3 independent experiments). * Significance for *p* < 0.05.

The three EOs were successfully used as an oily matrix component of NLC and the biocompatibility of the resulting nanosystems was also tested. A431 and HaCaT cells were exposed to test solutions containing the amount of NLC that gives equivalent EO concentrations as those tested as free EOs, and results are shown in Figure 3. Results show a similar behaviour of the treatment with LNLC and RNLC in both HaCaT and A431 cells, with IC_50_ values of about 0.075% (*v*/*v*) for HaCaT and about 0.114–0.117% (*v*/*v*) for A431 cells (Table 3). Those NLC systems showed a greater safety compared to ONLC, which negatively affected the cytotoxicity of HaCaT cells (Figure 3E vs. Figure 3B, and Table 3).

We previously found that Mediterranean EOs loaded-NLC were able to reduce the cytotoxicity of the pure EO on RAW 264.7 cells (murine macrophage cell line) [15]. Herein we found, interestingly, an increase in the ability of the EO in reducing the skin cell viability when it was encapsulated in the NLC structure (Table 3). This could be due to the presence of the surfactants–solid lipid membrane acting, at the nanoparticle surface, as a protective shell able to avoid the loss of the volatile compounds, thus increasing the bioavailability of EO to the biological systems [39].

Based on the presented data, NLC prepared with *Lavandula* and *Rosmarinus* (50 ng/mL) may be regarded as anti-proliferative agents and have potential to be used as co-adjuvants in the treatment of non-tumoral proliferative dermal diseases, such as infections, psoriasis, eczema, ichthyosis.

### 3.3. CLZ-Loaded NLC Prepared with the Selected EOs: Lavandula and Rosmarinus

**Figure 4:**
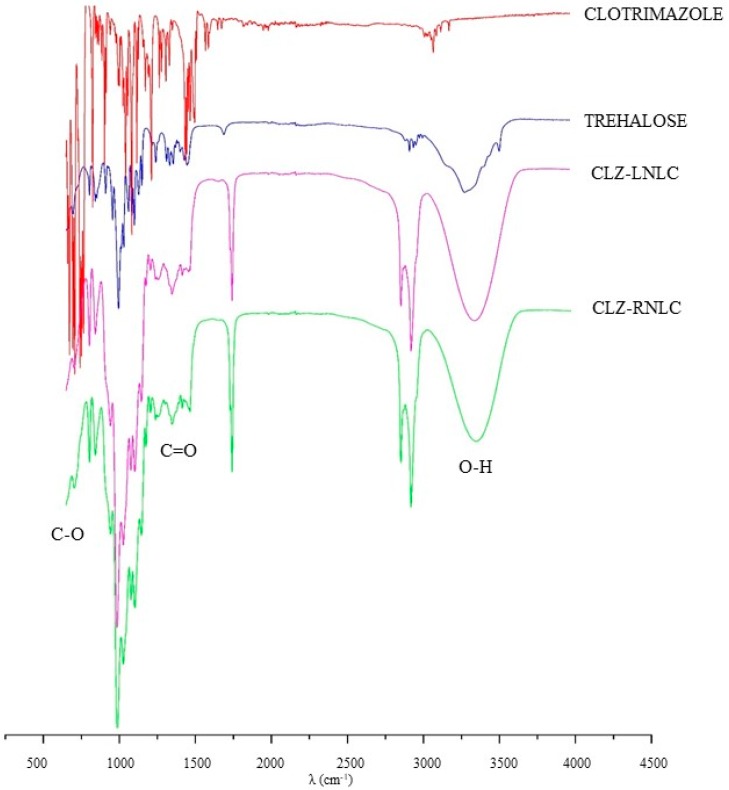
Fourier Transform-Infrared (FT-IR) spectra of pure clotrimazole (CLZ), cryoprotectant (Trehalose), CLZ-loaded NLC prepared with *Lavandula* and *Rosmarinus* EO (CLZ-LNLC, CLZ-RNLC).

Based on the obtained results on EOs-NLC, the optimized formulations prepared with *Lavandula* and *Rosmarinus* were selected for CLZ delivery. As reported in Table 4 and in Supplementary Figure S2, drug loading induced a 2-fold increase of mean particles size without affecting their homogeneity. A greater CLZ incorporation capability in NLC prepared with *Lavandula* EO was shown in respect to RNLC. However, the low values found for the EE% could be due to steric hindrance imparted to CLZ because of the spatial orientation of the phenyl rings, thus limiting not only the in vitro performance as reported by Sabzevari et al., but also the interactions with other components during the formation of NLC [40]. As previously reported, the disappearance of the characteristic absorption bands for a drug after incorporation into lipids, revealed by the FT-IR analysis, indicates the drug has transferred to the amorphous state [41]. Spectra of raw materials (surfactants and lipidic components) used to prepare NLC confirmed literature data (data not reported) [15]. Herein, CLZ as pure drug showed dominant absorption peaks at 1573.3, 1483.9 and 1315.2 cm^−1^, corresponding to the benzene ring stretches; peaks at 906.2, 821.6 and 756.3 cm^−1^ correspond to C–H stretches; bands at 1082.0 and 1206.2 cm^−1^ corresponding to chlorobenzene and C–N stretching, respectively (Figure 4). This data was in accordance with literature findings [42].

The peaks observed in CLZ powder spectrum, cannot be observed in the spectra of CLZ-loaded NLC, as some peaks of CLZ may overlap with the peaks from functional groups of the lipid and the EOs [42]. Indeed, Softisan 100 showed main characteristic absorption bands at 2800–2955 cm^−1^ (C–H stretching of benzene), 1728, 1739 cm^−1^ (carbonyl compound CHO), 1000–1300 cm^−1^ (C–O stretching), as we previously reported [15]. In our previous work we found that *Lavandula* and *Rosmarinus* EOs characteristic bands were in the range 3200–3600 cm^−1^ (O–H stretch), range 3000–3100 cm^−1^ (C–H stretching), 1670–1820 cm^−1^ (C=O), range 1400–1450 cm^−1^ (bending vibrations of CH_2_ and CH_3_ groups), range 1000–1330 cm^−1^ (C–O–C) and range 675–1000 cm^−1^ (C–H bending) [15]. Thus, FT-IR spectra obtained for the pure CLZ and CLZ-loaded NLC systems confirmed the drug was well incorporated into the nanoparticles prepared with *Lavandula* and *Rosmarinus* (Figure 4).

**Figure 5:**
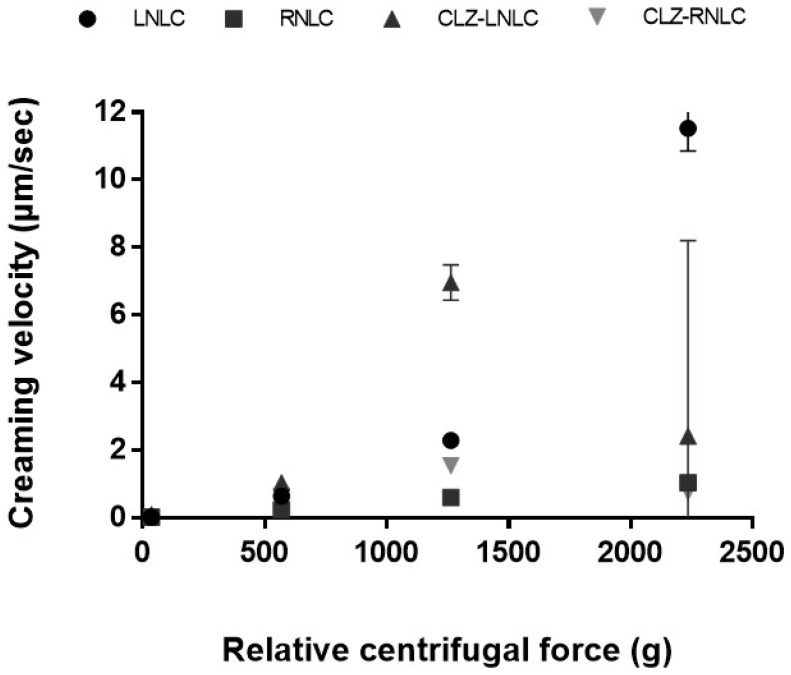
Speed of creaming at normal gravity obtained by LUMiSizer^®^ for unloaded and CLZ-loaded NLC prepared with *Lavandula* and *Rosmarinus* EO.

In order to confirm the stability of the optimized NLC, we exploited the dispersion analyzer LUMiSizer^®^. The innovative technology offers the possibility to achieve more time-saving information on sample stability (in terms of particles separation) compared to the traditional visual observation tests that take extensive period to determine the long-term stability of a colloidal suspension. LUMiSizer^®^ is able to detect fast stability ranking and shelf-life estimation of undiluted dispersions at their original concentration, in minutes/hours instead of months/years, exploiting centrifugal force to accelerate the occurrence of instability phenomena (sedimentation, flocculation or creaming) [43]. Considering the evolution of the obtained transmission profiles, the stability of the samples can be evaluated. Indeed, under centrifugal force, a stable colloidal dispersion allows the formation of a regular line, while aggregated particles show a typical step-profile, since the centrifugal acceleration induces different sedimentation speeds of particles with different diameter. In this work, the long-term stability of unloaded and CLZ-loaded NLC prepared with *Lavandula* and *Rosmarinus* EO was evaluated measuring the nanoparticles migration phenomena at high RCF, thus obtaining the creaming velocity by tracking the front movements at a specific transmittance with time (Figure 5). As reported in Figure 5, the stability of the NLC formulations decreases in the order: CLZ-RNLC > RNLC > CLZ-LNLC > LNLC. In particular, both unloaded and CLZ-loaded NLC prepared with *Rosmarinus* EO showed a greater stability compared to the NLC prepared with *Lavandula* EO that showed an increase in creaming velocity directly dependent on the RCF. As we previously reported, different oils are able to differently interact with other components in the formation of drug delivery systems [44,45]. Thus, it is possible that different EOs are able to induce the formation of NLC with different features, which may influence the stability of the colloidal dispersion. This hypothesis should be further investigated in the future, in order to deeper understand NLC features from a morphological point of view.

**Figure 6:**
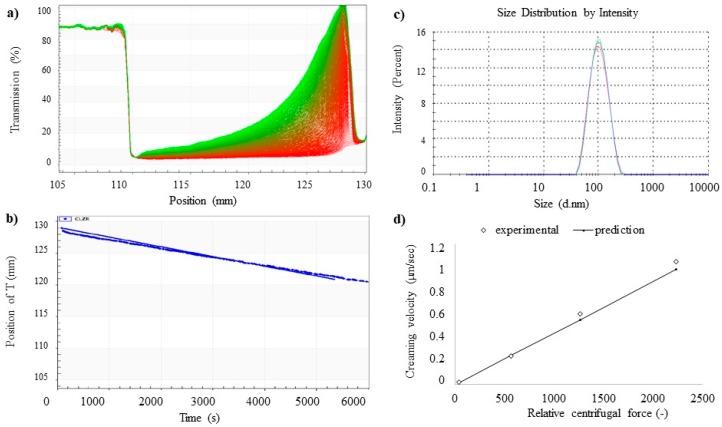
Creaming velocity prediction of CLZ-RNLC. (**a**) Evolution of transmission profiles at 3000 RCF. (**b**) Volume based size distribution of CLZ-RNLC suspension. (**c**) Transient variation of the front position in panel (**a**). The fitter straight line is determined as the creaming velocity. (**d**) Comparison of the measured creaming velocities and the theoretical values from Stoke’s law at different RCFs.

In order to achieve more information on CLZ-RNLC long-term stability, we compared results of the creaming velocity obtained with LUMiSizer^®^ at high RCF with the theoretical model (Stoke’s law), as previously reported by Chang et al. [27]. In Figure 6a the transmission profile across CLZ-RNLC sample in the analytical centrifuge recorded at a given time step during the centrifugation is depicted. The creaming velocities were calculated by Stoke’s law, using the experimental mean nanoparticles size obtained by PCS measurements (Figure 6b). The front of the creaming phenomenon was tracked with the time, and the slope was calculated as the creaming velocity (Figure 6c). Plotting the creaming velocity at different RCF, we obtained a linear increase with the value of RCF, thus confirming that particles migration instability phenomenon follows Stoke’s law. In addition, the closeness between the actual values measured for CLZ-RNLC to the predicted creaming velocities calculated by Stoke’s law using the mean nanoparticles diameter obtained by PCS, confirms that the instability phenomenon could be described by this theoretical model and can be used to extrapolate the creaming velocity at regular gravity of 1 RCF.

The results obtained by the accelerated stability studies, allowed us to establish the potential shelf life of the prepared NLC colloidal suspension, according to the highest industry norms and regulations ISO 13318-1 and ISO 13318-2 (concerning nanoparticle accelerated stability studies). In particular, since the nanoparticle separation, due to the occurrence of creaming phenomena, was not relevant for sample RNLC and CLZ-RNLC, we could reasonably estimate a shelf life of 12 months at 25 °C, at least. It is worth noting that the obtained stability results were in perfect agreement with PCS measurements of samples analyzed after 2 and 6 months of storage, showing the absence of significant variations in mean size and polydispersity values (Supplementary Table S1).

**Figure 7:**
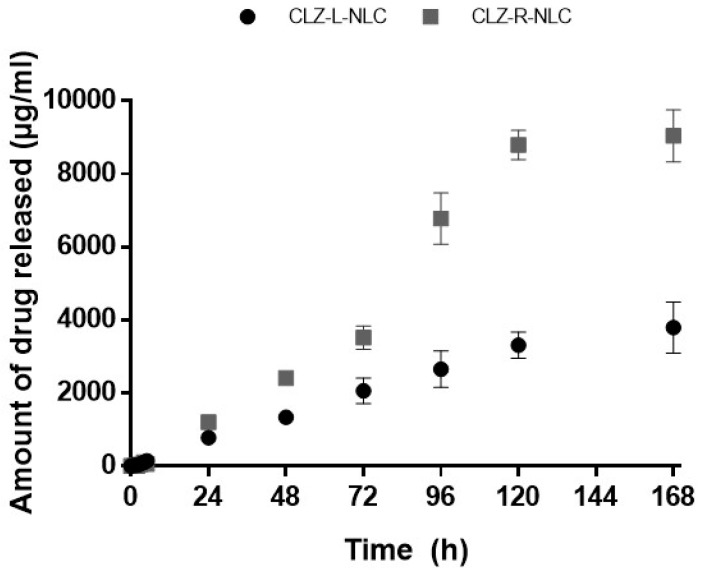
Amount (µg/mL) of CLZ released in vitro from NLC prepared with *Lavandula* EO and *Rosmarinus* EO.

As reported in literature, increasing resistant phenomena have been developed against *Candida* species [1,4,46]. In order to overcome resistance against CLZ, we exploited drug nanoencapsulation in Mediterranean EOs-NLC, using *Lavandula* or *Rosmarinus* EO as potential synergistic oily components. In vitro release profiles of CLZ from the prepared NLC are shown in Figure 7. NLC systems were able to promote a prolonged CLZ release, without any initial burst effect, thus confirming drug incorporation into the inner core of nanoparticles, without residual amount of drug absorbed on their surface. These results are in accordance with literature findings concerning drug release profile from NLC. Indeed, as we previously demonstrated, the presence of the liquid oily component in the solid lipid matrix allows the formation of irregular spaces in which the drug can be better accommodated, thus providing a controlled drug delivery [44].

It is worth noting that even if *Lavandula* EO allowed the encapsulation of a greater amount of CLZ (45.81 mg) into the NLC compared to *Rosmarinus* (25.12 mg), it promoted a slower drug release compared to CLZ-RNLC. Indeed, after 24 h a similar amount of CLZ was released from both formulations (almost 4 mg), but after 120 h from the beginning of the experiment, less than 10% of CLZ was released from LNLC while RNLC released almost 40% of the encapsulated drug. These data demonstrate the possibility to prepare lipid nanoparticles with different release profiles, depending on the selected EO used as oily matrix component. In particular, the interesting behavior of CLZ-RNLC is not only related to its ability to provide a sustained prolonged CLZ release, but also to the possibility to economize the amount of the loaded drug, determining the formation of a nanosized colloidal suspension characterized by a long-term physical stability, as previously demonstrated.

### 3.4. Pre-Vitro Evaluation of Essential Oils on Biomembrane 

In order to characterize the potential mechanisms related to cytotoxicity effects, *Lavandula* and *Rosmarinus* as pure EO were investigated for their potential capability to interact with model membranes through calorimetric technique. Each oil is characterized by a mixture of different components that influence biomembrane structure [15]. It is known that compound–membrane interaction is a dynamic phenomenon, influenced by different factors such as compound’s chemical structure and membrane organization. Furthermore, it can also be affected by compound internalization and absorption at the membrane–water interface. 

**Figure 8:**
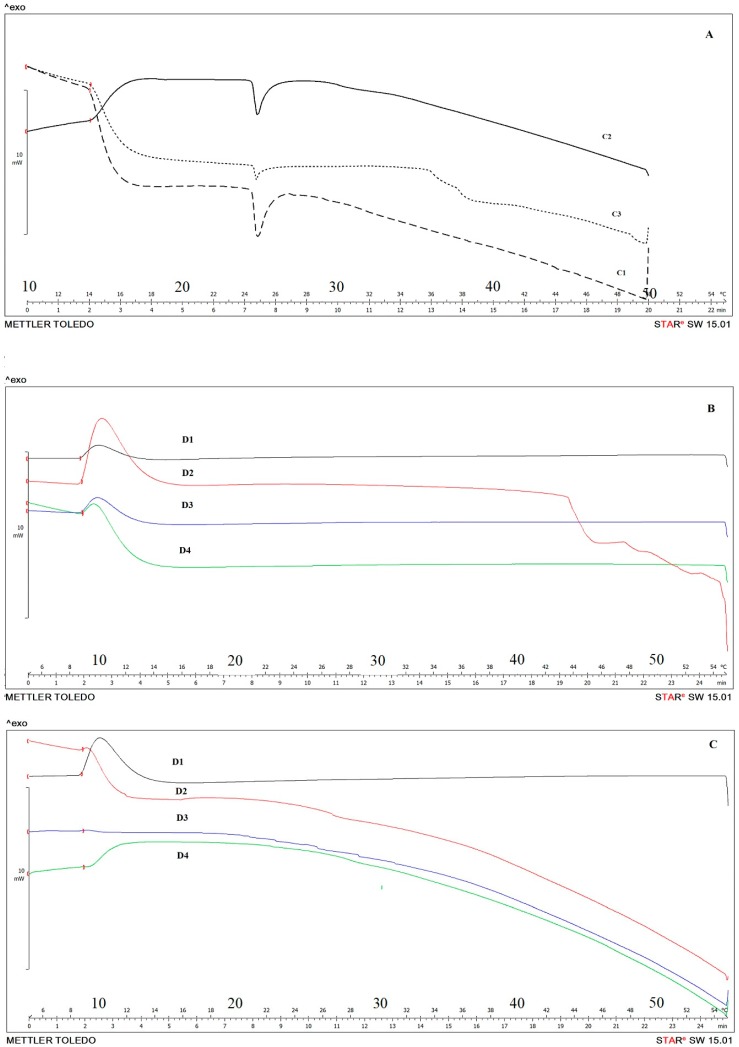
Thermograms of MLV at different dilutions C1, C2, C3 respectively 0.05%, 0.1%, 0.2% (*v*/*v*) (**A**), MLV with *Lavandula* (**B**) and *Rosmarinus* (**C**). EOs were tested at different dilutions: D1, D2, D3, D4 corresponding to 0.01%, 0.05%, 0.1%, 0.2% *v*/*v* respectively.

In Figure 8A we reported the calorimetric curves related to MLV obtained with different dilutions, corresponding to the same used for interaction studies with EOs. Except for the lowest dilution, all tested amount of MLVs did not influence the T_M_ rather than the enthalpy values of the main transition peak of DMPC bilayers (Supplementary Table S2). The EOs concentrations chose for DSC study were selected based on the results obtained in the in vitro cytotoxicity test. 

As previously demonstrated by Cristani et al., antimicrobial activity of some compounds may be the result of the perturbation of the lipidic fraction of the microorganism plasmic membrane [47]. As reported in Figure 8B,C, both tested EOs induced the disappearance of the main transition peak demonstrating their capability to dissolve in the aqueous medium and interact with lipidic bilayers. 

These results suggest that the ability of *Lavandula* and *Rosmarinus* to interact with the membrane permeabilization, with the consequent perturbation of the lipid fraction membrane, can be exploited to the advantage of a potential antifungal activity. Indeed, the potential antifungal activity may be due to the migration of components across the aqueous extracellular medium, interacting with the lipidic membranes with a consequent damage [48].

### 3.5. In Vitro Antifungal Susceptibility Test: MIC and MLC Results

Based on the encouraging results obtained testing EOs with model membranes, preliminary studies on the antifungal activity of CLZ-loaded EO-NLCs were performed, evaluating the susceptibility of three *Candida* spp. strains to these formulations. In order to achieve a proper understanding of the effect of each component, loaded and unloaded EO-NLCs were tested, as well as the free drug and the pure EOs. The tested formulations, as colloidal suspensions, presented natural turbidity even after several dilutions, thus we the OD screening measurements after the microplate incubation were very inconclusive. Therefore, the content of the first five dilutions of each formulation was transferred to PDA plates. Colonies formed after 24 h of incubation at 35 °C were counted in order to monitor yeast growth and compared to the starting count of the inoculum. Our observations showed that the concentration values that killed the yeast inoculum (no colonies growth and counted) and the immediate lower concentration value allowed the growth of the inoculum (no increase on cell counting). Thus, we considered that the MIC and MLC values were the same for each formulation within the tested concentration ranges. The MICs and MLCs determined for *C. albicans* ATCC 10231, *C. krusei* ATCC 6258 and *C. parapsilosis* ATCC 90098 are summarized in Table 5.

A primary overview at these results shows that *C. parapsilosis* is the most sensitive strain to the majority of the tested solutions, while *C. krusei* seems to be the most resistant one among the three reference strains. Regarding the tested pure EOs (*Lavandula* and *Rosmarinus*), although little information is provided in the literature, the MICs and MLCs determined for each strain are in accordance to previous findings by Bona et al. [8] within the range of 2–4% (*v*/*v*) for both EOs in reference and clinical isolated strains of *C. albicans*. Interestingly, *Lavandula* as pure EO was found to be more active than *Rosmarinus* EO (Table 5). This could be probably due to the highest amounts of terpenes in *Lavandula* EO (linalool 30% and linalyl acetate 38%) compared to *Rosmarinus* (1,8 cineol 50%), whose antifungal activity against several strains of *Candida*, including *C. albicans*, *C. glabrata*, and *C. parapsilosis*, have been previously reported [49]. However, conflicting results are described in literature concerning *Rosmarinus* antifungal activity, such as the lack of anti-candidal activity of *R. officinalis* L. samples collected from National Parquet El Hamma (Algiers) reported by Djeddi et al. [50]. Thus, it is possible that geographic factors, in addition to seasonal ones, strongly affect the variability of the antifungal activity of this plant, as properly suggested by Ksouri et al. [51]. Our findings also demonstrated that the antifungal activity of both EOs is retained when they are loaded into the NLC formulations (Table 5), probably on account of the elevated stability of the lipid matrix that prevents EO dispersion within the exterior medium or within the yeast cells, if cellular internalization is considered. Interesting results were obtained for CLZ-loaded NLC, as the co-existence of drug and EO lead to an improvement of the antifungal activity. As reported by Langevel et al., different studies presented the occurrence of a moderate synergism between EOs and drugs such as antibiotics (most likely due to membrane interactions of the EO compounds), in which case EOs provide an interesting option to reduce the use of antibiotics [52]. In particular, CLZ-LNLC provides an increase of the antifungal activity of *Lavandula* EO of four-fold for *C. albicans*, two-fold for *C. krusei* and eight-fold for *C. parapsilosis*, while CLZ-RNLC provides an increase of the antifungal activity of the *Rosmarinus* EO of eight-fold for *C. albicans*, four-fold for *C. krusei* and four-fold for *C. parapsilosis*. Moreover, the antifungal activity of free CLZ is also increased when loaded into both EO-NLCs for *C. albicans* and *C. parapsilosis*.

## 4. Conclusions

Taken all together, our results allow us to infer that the nanoencapsulation of the selected antifungal drug into NLC systems prepared using Mediterranean EOs as intrinsic oily components represents a promising strategy to improve CLZ effectiveness against candidiasis. In particular, our results open the debate concerning the possibility to exploit the intrinsic properties of *Lavandula* and *Rosmarinus*, whose synergistic effects are to be further investigated, and could offer a strategy in using CLZ-EO-NLC for overcoming drug resistance mechanisms involved in the treatment of topical infections.

## Figures and Tables

**Table 1:** Parameters of the response surface for mean particles size (Zave) and polydispersity (PDI) obtained from a 2^2^ full factorial design. β1 and β2 are the coefficients of the independent variables, β0 is the arithmetic mean response, β12 is the interaction term.

Investigated Parameters	β0	β1	β2	β12
**Zave**				
**Effect**	100	−91.67	−5.64	14.56
**Standard error**	5.26	13.91	13.91	13.91
**Significance level**	25.21	−6.59	−0.41	1.05
**t value**	0.0001	0.007	0.71	0.37
**PDI**				
**Effect**	0.16	−0.12	0.11	0.11
**Standard error**	0.009	0.025	0.025	0.025
**Significance level**	17.35	−4.91	−4.32	4.20
**t value**	0.0004	0.01	0.025	0.025

**Table 2:** ANOVA parameters for the characterization of the 2^2^ full factorial design.

Investigated Parameters	Sum of Squares (SS)	Mean of Squares (MS)	F	Significance *p* Value
**Zave**				
**A**	84.04 10^2^	84.04 10^2^	43.40	0.007 *
**B**	31.81	31.81	0.16	0.712
**AB**	211.99	211.99	1.09	0.372
**Error**	580.81	193.60		
**Total SS**	92.28 10^2^			
**PDI**				
**A**	1.54 10^−2^	1.54 10^−2^	24.17	0.016 *
**B**	1.19 10^−2^	1.19 10^−2^	18.67	0.022 *
**AB**	1.12 10^−2^	1.12 10^−2^	17.66	0.024 *
**Error**	0.19 10^−2^	0.000636		
**Total SS**	4.04 10^−2^			

* Significance for *p* < 0.05.

**Table 3:** Results of cell viability, expressed as IC50 (concentration that inhibits 50% of cell growth), for HaCaT and A431 cells, exposed for 24 h to different concentrations (% *v*/*v*) of essential oils (EO) (*Lavandula*, *Origanum* and *Rosmarinus*) and of respective nanostructured lipid carriers (NLC). The NLC were composed of 4% (*v*/*v*) respective EO and concentration of NLC was set as EO equivalent (see methods for details).

Cell Line	Sample	*Lavandula*	*Origanum*	*Rosmarinus*
**HaCaT**	EO	0.228 ± 0.004	0.022 ± 0.001	0.258 ± 0.003
	NLC	0.076 ± 0.001	0.012 ± 0.001	0.075 ± 0.001
**A431**	EO	0.274 ± 0.006	0.012 ± 0.001	0.263 ± 0.002
	NLC	0.114 ± 0.003	0.016 ± 0.001	0.117 ± 0.003
**Effect of significance for skin cell lines**	EO vs. NLC for HaCaT Significant	EO vs. NLC for A431 Significant	HaCaT vs. A431 for EO Significant	HaCaT vs. A431 for NLC Significant
	*p* < 0.0001	*p* < 0.0001	*p* < 0.0001	*p* < 0.0001

**Table 4:** Mean particle size (Zave, nm), polidispersity index (PDI) and percentage of encapsulation efficiency (EE%) ± standard deviation (S.D.) of unloaded and clotrimazole (CLZ) loaded NLC prepared using *Lavandula* (L) and *Rosmarinus* (R) essential oil. Each value is the average of six different experiments.

Samples	Zave (nm) ± S.D.	PDI ± S.D.	EE% ± S.D.	LC ± S.D.
**LNLC**	85.89 ± 0.51	0.113 ± 0.018	-	-
**RNLC**	76.97 ± 0.51	0.116 ± 0.012	-	-
**CLZ-LNLC**	163.0 ± 3.32	0.164 ± 0.025	25.2 ± 1.02	96.74 ± 0.5
**CLZ-RNLC**	126.8 ± 2.83	0.173 ± 0.008	16.7 ± 2.06	97.89 ± 0.6

**Table 5:** Antifungal activity Minimum inhibitory concentration (MIC) and minimum lethal concentration (MLC) of free CLZ, pure EOs, unloaded and CLZ-loaded EO NLC for *Candida* strains (mean values; *n* = 3).

Sample	Strains	*C. albicans* ATCC 10231	*C. krusei* ATCC 6258	*C. parapsilosis* ATCC 90098
**CLZ**	MIC^+^	>128	>128	64
	MLC^+^	>128	>128	64
***Pure Lavandula***	MIC^†^	0.5	0.5	0.5
	MLC^†^	0.5	0.5	0.5
***Pure Rosmarinus***	MIC^†^	2	2	0.5
	MLC^†^	2	2	0.5
**LNLC**	MIC^†^	1	>2	>2
	MLC^†^	1	>2	>2
**RNLC**	MIC^†^	>2	>2	>2
	MLC^†^	>2	>2	>2
**CLZ-LNLC**	MIC^‡^	0.125 + 78	0.25 + 156	0.0625 + 39
	MLC^‡^	0.125 + 78	0.25 + 156	0.0625 + 39
**CLZ-RNLC**	MIC^‡^	0.25 + 62.5	0.5 + 125	0.125 + 31.25
	MLC^‡^	0.25 + 62.5	0.5 + 125	0.125 + 31.25

^+^ Results expressed in CLZ μg mL^−1^ (*m*/*v*); ^†^ Results expressed in EO% (*v*/*v*); ^‡^ Results expressed in EO% (*v*/*v*) + CLZ μg mL^−1^ (*m*/*v*).

## Supplementary Materials

The following are available online at https://www.mdpi.com/1999-4923/11/5/231/s1: Figure S1. Thermograms and temperature of crystallization peak of pure EOs during a heat scan in the range of temperature 5–55 °C: *Rosmarinus* (R.O.), *Lavandula* (L.O.), *Origanum* (O.O.). Figure S2. Mean size distribution measured as percentage of scattered light of unloaded LNLC (a) and RNLC (b) and clotrimazole-loaded CLZ-LNLC (c) and CLZ-RNLC (d). Table S1. Mean particle size (Zave, nm) and polidispersity index (PDI) ± standard deviation (S.D.) of unloaded and clotrimazole (CLZ) loaded NLC prepared using *Lavandula* (L) and *Rosmarinus* (R) EO and analyzed after 2 and 6 months of storage at 25 °C. Each value is the average of six different experiments. Table S2. Thermotropic parameters of mesophase transition from gel (Lβ) to liquid crystalline phase (Lα) of multilamellar vesicle dispersions made up of DMPC at different dilutions.
