# Supplementary material for: Clotrimazole-Loaded Mediterranean Essential Oils NLC: A Synergic Treatment of Candida Skin Infections
Source: Pharmaceutics. 2019 May 13;11(5):231. doi: 10.3390/pharmaceutics11050231 (PMC6572383; doi:10.3390/pharmaceutics11050231)

a)

			Size (d.nm...	% Intensity:	St Dev (d.n...
Z-Average (d.nm):	85.91	Peak 1:	95.96	100.0	32.31
PdI:	0.107	Peak 2:	0.000	0.0	0.000
Intercept:	0.953	Peak 3:	0.000	0.0	0.000
Result quality	Good				

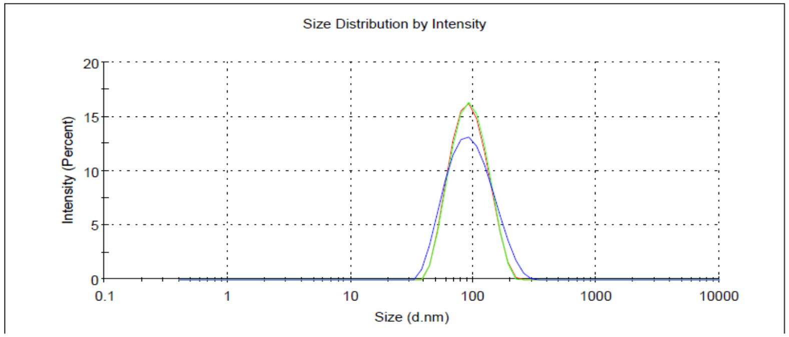

b)

			Size (d.nm...	% Intensity:	St Dev (d.n...
Z-Average (d.nm):	76.41	Peak 1:	86.14	100.0	30.73
PdI:	0.104	Peak 2:	0.000	0.0	0.000
Intercept:	0.964	Peak 3:	0.000	0.0	0.000
Result quality	Good				

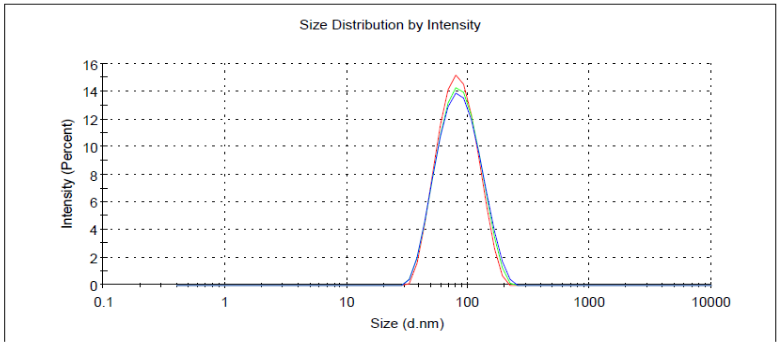

c)

			Size (d.nm...	% Intensity:	St Dev (d.n...
Z-Average (d.nm):	163.8	Peak 1:	184.4	100.0	62.03
PdI:	0.101	Peak 2:	0.000	0.0	0.000
Intercept:	0.970	Peak 3:	0.000	0.0	0.000
Result quality	Good				

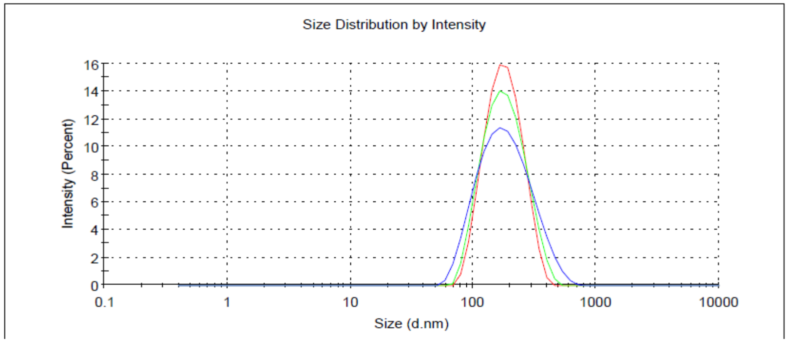

d)

			Size (d.nm...	% Intensity:	St Dev (d.n...
Z-Average (d.nm):	125.4	Peak 1:	148.5	100.0	67.85
PdI:	0.175	Peak 2:	0.000	0.0	0.000
Intercept:	0.951	Peak 3:	0.000	0.0	0.000
Result quality	Good				

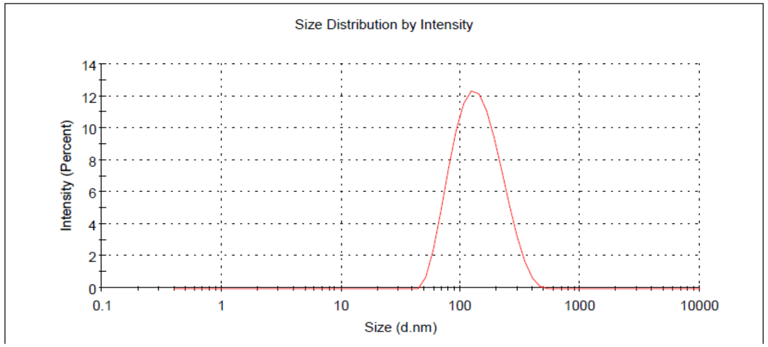

Supplement: Supplementary file 1 [file pharmaceutics-11-00231-s001.zip › Supplementary Figure S2.pdf]
