# Supplementary material for: Clotrimazole-Loaded Mediterranean Essential Oils NLC: A Synergic Treatment of Candida Skin Infections
Source: Pharmaceutics. 2019 May 13;11(5):231. doi: 10.3390/pharmaceutics11050231 (PMC6572383; doi:10.3390/pharmaceutics11050231)
Supplement: Supplementary file 1 [file pharmaceutics-11-00231-s001.zip › Supplementary Tables.pdf]

Supplementary Table 1. Mean particle size (Zave, nm) and polidispersity index (PDI) ± standard deviation (S.D.) of unloaded and clotrimazole (CLZ) loaded NLC prepared using *Lavandula* (L) and *Rosmarinus* (R) essential oil and analyzed after 2 and 6 months of storage at 25 °C. Each value is the average of six different experiments.

Time Storage	Samples	Zave (nm) ± S.D.	PDI ± S.D.
2 months	LNLC	88.0 ± 2.2	0.095 ± 0.006
	RNLC	77.6 ± 1.1	0.140 ± 0.023
	CLZ-LNLC	159.7 ± 2.7	0.120 ± 0.003
	CLZ-RNLC	137.2 ± 1.9	0.094 ± 0.010
6 months	LNLC	102.0 ± 1.5	0.114 ± 0.010
	RNLC	87.6 ± 4.9	0.140 ± 0.014
	CLZ-LNLC	162.0 ± 1.4	0.176 ± 0.016
	CLZ-RNLC	139.6 ± 4.0	0.153 ± 0.031

Supplementary Table 2. Thermotropic parameters of mesophase transition from gel (Lβ) to liquid crystalline phase (Lα) of multilamellar vesicle dispersions made up of DMPC at different dilutions.

Sample	Tm °C	ΔH (Jg)
MLV	25.18±0.02	-48.32±0.20
C1	24.89±0.05	-44.46±0.31
C2	24.68±0.07	-28.61±0.26
C3	25.22±0.03	-20.13±0.23
