# Supplementary figures and images for: Clotrimazole-Loaded Mediterranean Essential Oils NLC: A Synergic Treatment of Candida Skin Infections
Source: Pharmaceutics. 2019 May 13;11(5):231. doi: 10.3390/pharmaceutics11050231 (PMC6572383; doi:10.3390/pharmaceutics11050231)

exo

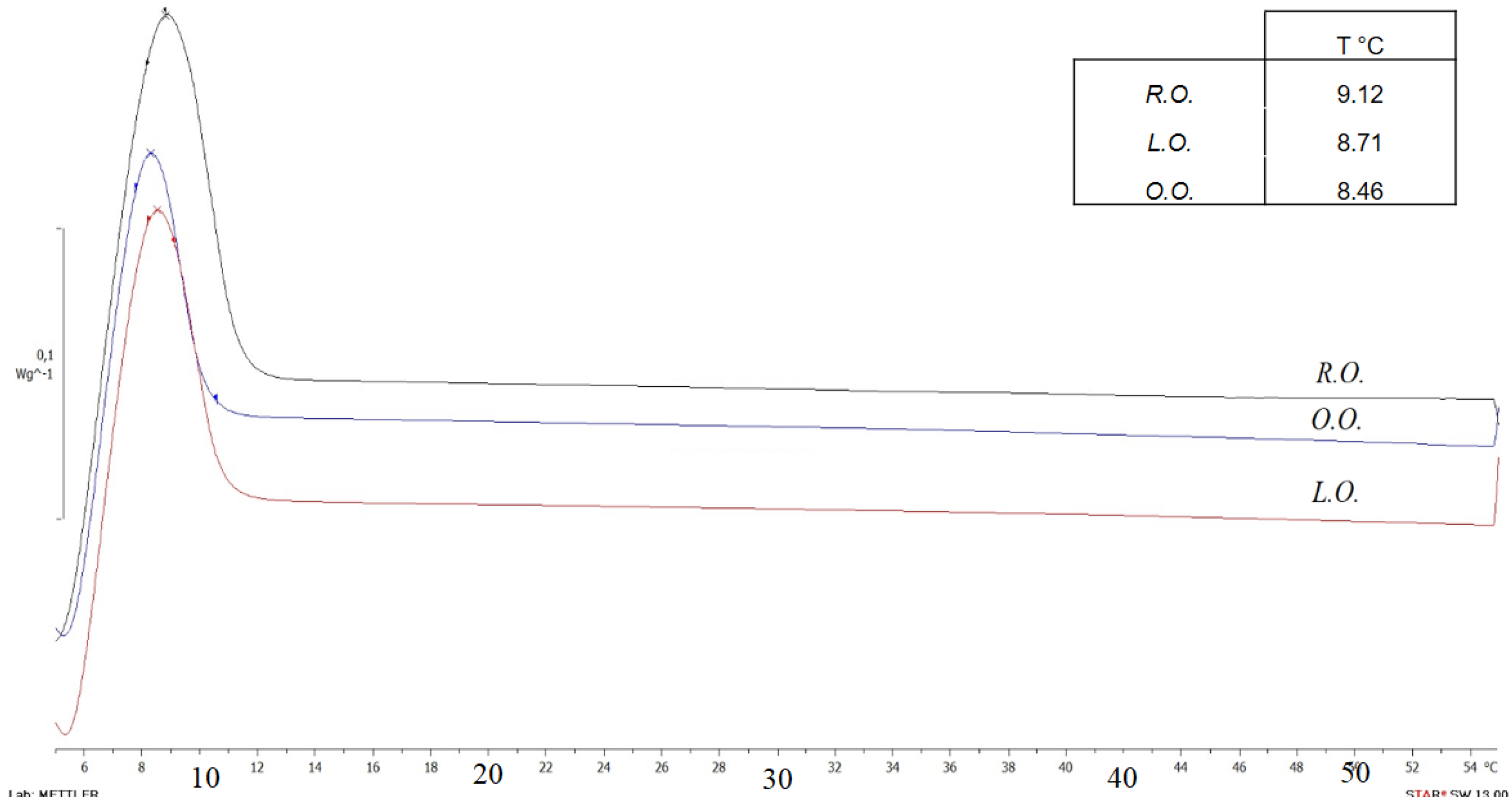

|             | T °C |
|-------------|------|
| <i>R.O.</i> | 9.12 |
| <i>L.O.</i> | 8.71 |
| <i>O.O.</i> | 8.46 |

Supplement: Supplementary file 1 [file pharmaceutics-11-00231-s001.zip › Supplementary Figure S1.pdf]
